# Clinical and pathological analysis of intravascular large B-cell lymphoma of the prostate

**DOI:** 10.1007/s12672-025-04141-3

**Published:** 2025-11-25

**Authors:** Jiangying Zhao, Lu Li, Jia Yang, Jiao Peng, Chunlin Li

**Affiliations:** 1 Department of Pathology, Mianyang Hospital of T.C.M, Mianyang, 621000 Sichuan People’s Republic of China; 2 Department of Urology, Mianyang Hospital of T.C.M, Mianyang, 621000 Sichuan People’s Republic of China

**Keywords:** Prostate, Benign prostatic hyperplasia, Diffuse large B-cell lymphoma, Intravascular large B-cell lymphoma

## Abstract

**Background:**

Intravascular large B-cell lymphoma is a rare type of lymphoma. Although this tumor can occur in several parts of the body, primary intravascular large B-cell lymphoma of the prostate is extremely rare. Currently, there are only four case reports of primary large B-cell lymphoma in the prostate, and there is no comprehensive analysis of case series. Here, we report a case of intravascular large B-cell lymphoma originating from the prostate. Due to the unique morphology and atypical location of tumors under a microscope, pathologists often overlook them.

**Case presentation:**

A 52-year-old Chinese male presented with frequent urination, urgency, and nocturia. Magnetic resonance imaging (MRI) showed prostate hyperplasia, while biopsy revealed the hyperplasia of prostate glands and stroma. A small area had dilated blood vessels, and there were many proliferating cells in the lumen. The cell nucleus was large, the nuclear cytoplasmic ratio was small, and the nuclear morphology was irregular. Multiple immunohistochemical staining were negative, but CD20 was positive. No other tumors were detected on the patient’s whole body scan. We diagnosed it as intravascular large B-cell lymphoma of the prostate.

**Conclusion:**

In patients with benign prostate hyperplasia, proliferative blood vessels are found in the interstitium, and there is an accumulation of proliferative large cells in the lumen, which do not resemble endothelial cells, epithelial cells, tissue cells, or inflammatory cells. Pathologists should consider the possibility of intravascular large B-cell lymphoma and conduct immunohistochemical staining for large B-cell lymphoma. Primary intravascular large B-cell lymphoma of the prostate should be diagnosed after ruling out metastatic tumors.

## Introduction

Intravascular large B-cell lymphoma (IVLBCL) is a rare subtype of non-Hodgkin’s lymphoma, with an incidence rate of approximately 0.5 per 1,000,000 [1]. The most commonly affected organs are the skin [2], central nervous system [3], and bone marrow [4], but it may also affect other parts and organs [5, 6]. It is often accidentally discovered in other pathological examinations or presents with slowly growing painless masses. When the lesion is small, it can be easily mistaken for vasculitis, epithelial tumors, and other lesions. Intravascular large B-cell lymphoma (IVLBCL) is pathologically known for its unique pattern of tumor cell infiltration. Tumor cells of IVLBCL quietly gather and form lesions in the seemingly harmless blood vessels of ordinary people. The morphology and immune phenotype of these tumor cells are crucial for diagnosis. In prostate tissue biopsy, pathologists can find abnormal lymphocytes clustered in small blood vessels, appearing morphologically as atypical B cells, namely CD20 and CD79α-positive cells. Cell proliferation can be reflected using Ki67 immunohistochemical staining. Primary intravascular large B-cell lymphoma of the prostate is extremely rare, accounting for only 0.09% of all prostate tumors and 0.1% of all non-Hodgkin’s lymphomas (NHL) [7]. Through a review of the English literature, we found only four cases of primary intravascular large B-cell lymphoma originating from the prostate [8–11], and their clinical manifestations and genetic and epidemiological characteristics remain unclear. This article explored this unique pathological phenomenon in depth through case reports and literature review.

## Case presentation

**Fig. 1:**
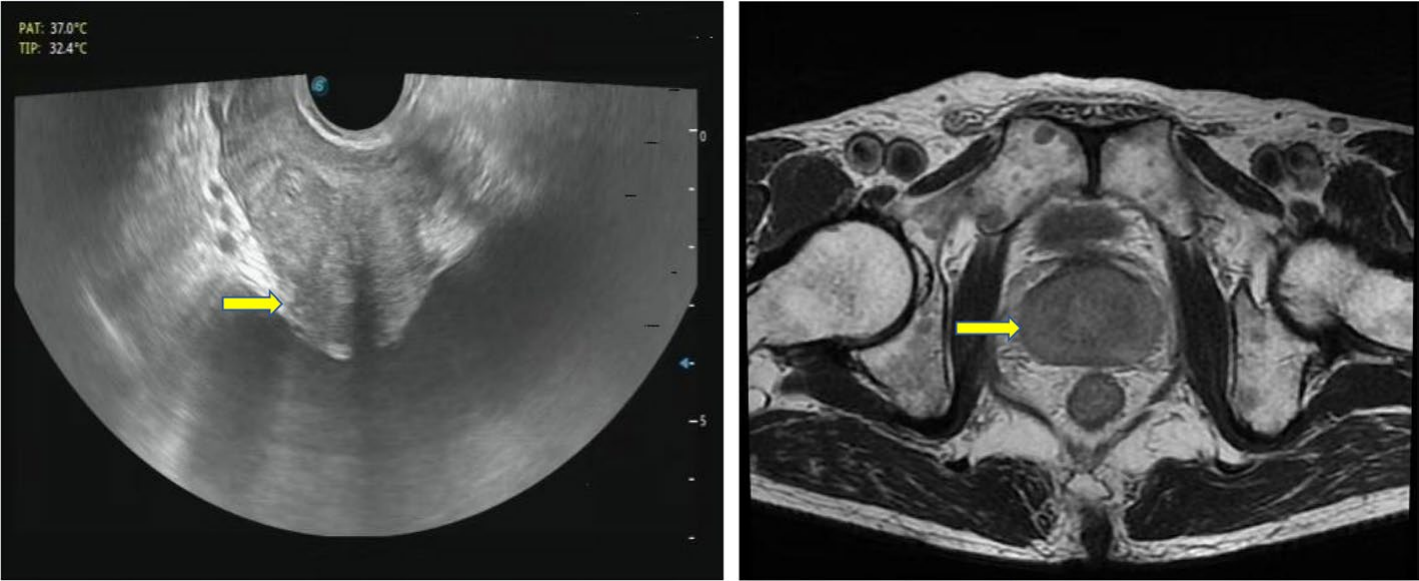
Prostate color ultrasound and magnetic resonance imaging (MRI) showed grade II prostate hyperplasia

**Fig. 2:**
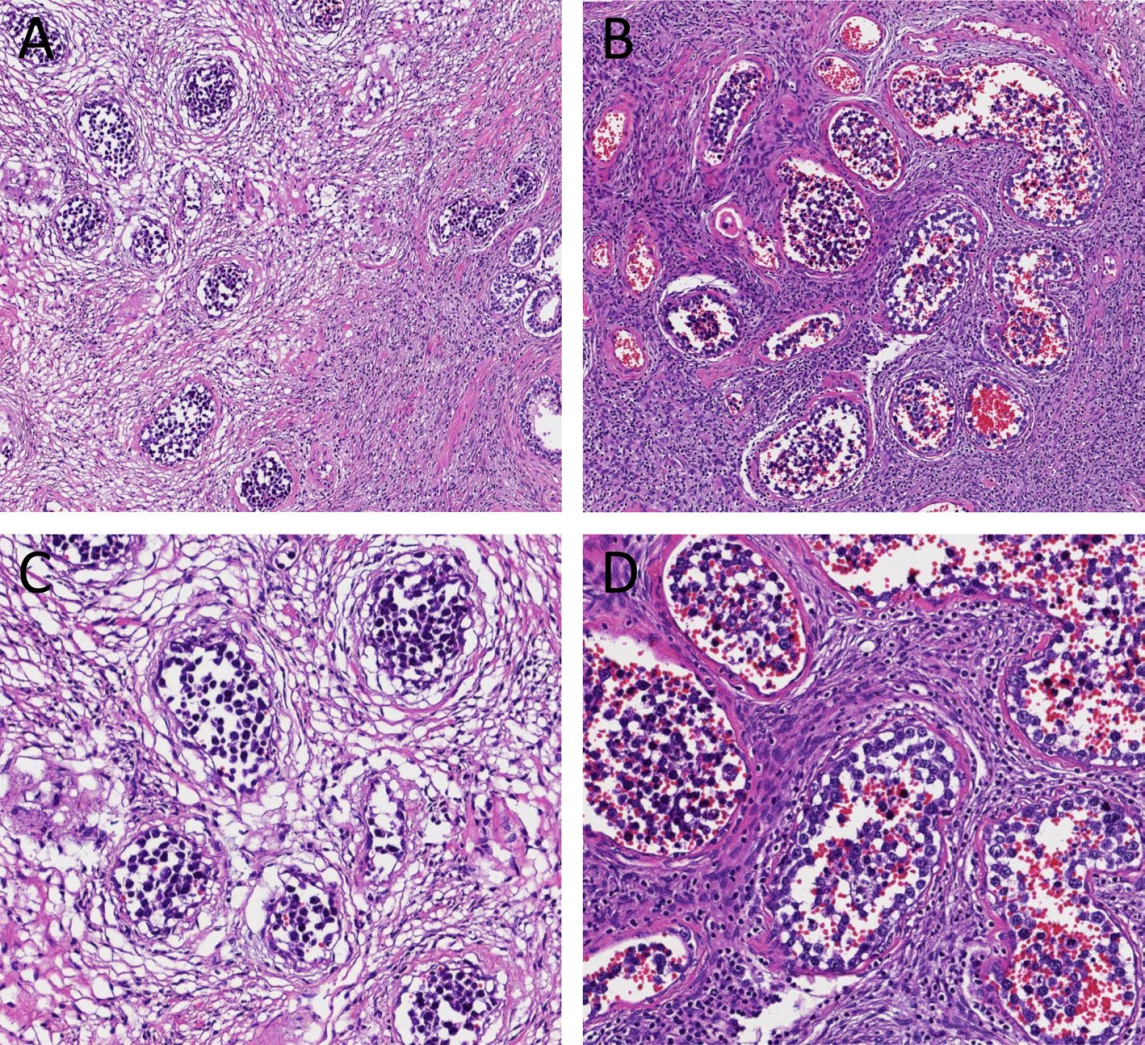
Histological features (hematoxylin and eosin stain). In a very small area of the prostate stroma, we found a small number of dilated blood vessels with abnormal cells distributed in the vascular lumen. The cell volume was large, the nuclear cytoplasmic ratio was high, the karyotype was irregular, the nucleus was deeply stained, and the cells were free in the vascular lumen. (**A** and **B**: H&E × 100; **C** and **D**: H&E × 200)

**Fig. 3:**
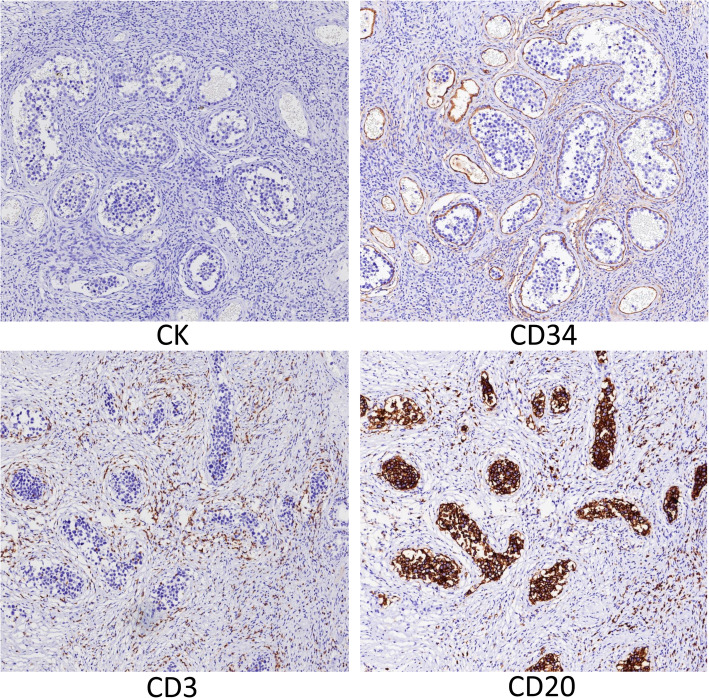
Immunohistochemical staining showed that the tumor cells expressed high levels of CD20, CD79a, PAX5, and Ki-67 (close to 90%), but CK, PSA, CD31, CD34, ERG, CD3, CD56, and TIA1 were all negative. (IHC × 100)

A 52-year-old Chinese male patient presented with progressive difficulty in urination. Prostate color ultrasound and magnetic resonance imaging (MRI) showed grade II prostate hyperplasia (Fig. 1), and prostate-specific antigen (PSA) level was normal. After transurethral resection of the prostate, the surgical specimen showed signs of prostate hyperplasia under the microscope, and there was no evidence of prostate tumors. However, in a very small area of the prostate stroma, we found a small number of dilated blood vessels with abnormal cells distributed in the vascular lumen. The cell volume was large, the nuclear cytoplasmic ratio was high, the karyotype was irregular, the nucleus was deeply stained, and the cells were free in the vascular lumen (Fig. 2). Initially, we considered them to be some shed endothelial cells or tissue cells. We also conducted immunohistochemical staining for PSA, AMACR, CD31, CD34, ERG, CD68, and CD163, but unfortunately, the results were all negative. Considering the structural characteristics of cells after HE staining, we conducted a second round of immunohistochemical staining to exclude the possibility of hematogenous metastasis of epithelial tumors in other parts of the body. This time, we added antibodies for epithelial tumor markers, such as CK, CK5/6, CK7, EMA, etc., but surprisingly, the staining results of these antibodies were still all negative. The patient was an elderly male who had no history of any tumors. We conducted a whole-body PET-CT scan and did not find any tumor lesions. Based on the cell morphology in HE staining, we still consider these intravascular cells as abnormal cells and suspect them to be tumor cells. However, immunohistochemical staining could not identify the histological type of the tumor and determine whether it is primary or metastatic. Abnormal cells grew inside blood vessels. We excluded epithelial cells, vascular endothelial cells, and tissue cells, and considered lymphocytes. However, abnormal lymphocytes grow inside blood vessels, and the patient had no history of lymphoma, surface or deep lymph node enlargement, and no abnormalities in the hematologic system. After ruling out tumors of the lymphatic and hematopoietic systems affecting the prostate, intravascular NK/T cell lymphoma and intravascular large B-cell lymphoma were suggested as the first diagnosis. After reviewing relevant literature, we found that primary intravascular NK/T cell lymphoma and intravascular large B-cell lymphoma can rarely occur in the prostate. The tumor morphology and structure in the literature were similar to this case. Therefore, we performed immunohistochemical staining for CD3, CD56, TIA1, CD20, CD79a, PAX5, and Ki-67 on these abnormal cells in the blood vessels. The tumor showed strong expression of CD20, CD79a, PAX5, and Ki-67 (close to 90%), but CD3, CD56, and TIA1 were all negative (Fig. 3). No tumor lesions were found in the patients’ body, and there was no history of tumor, lymph node enlargement, or hematopoietic system abnormalities. In addition, the metastasis of lymphoma was ruled out. Based on the possibility of prostate enlargement, we consider the elderly male patient with primary intravascular large B-cell lymphoma of the prostate. The patient received chemotherapy and was followed for 8 months. The therapeutic effect was satisfactory.

## Discussion

IVLBCL has been named due to its unique pathological features, where tumor cells mainly infiltrate into blood vessels. This rare cellular localization makes it unique in the lymphoma lineage. Its clinical manifestations are non-specific, often presenting with systemic symptoms and dysfunction of local organs [12]. The prostate receives abundant blood supply from the prostate artery, infravesical artery, and infrarectal artery, which makes it a suitable place for intravascular diseases, such as IVLBCL. In IVLBCL, tumor cells directly invade small blood vessels in the prostate, leading to vascular obstruction and impairing the normal physiological function of the prostate [13]. Due to the similarity between IVLBCL, BPH, and prostate cancer in terms of the location, clinical symptoms, and pathological manifestations, detailed medical history, imaging examination, histopathological staining, and immunohistochemical analysis are necessary to clarify the diagnosis and guide individualized treatment plans [14].

The patient in this case was admitted for the treatment of benign prostatic hyperplasia and underwent prostate resection. After surgery, abnormal cells were found in the blood vessels under the background of benign prostatic hyperplasia. Due to the small number of abnormal cells, some were distributed in blood vessels. Pathologists first diagnosed them as endothelial cells and tissue cells, but negative immunohistochemical staining for CD31, CD34, ERG, CD68, and CD163 did not support this diagnosis. The lesion was found in the prostate. Although no evidence of prostate cancer was found, it could not be ruled out that prostate cancer may have been missed during surgery or sectioning. Therefore, it was necessary to determine whether the cells in the blood vessels were prostate cancer cells. However, PSA and AMACR staining were still negative and ruled out prostate cancer. The abnormal cells in the blood vessels had a large volume, similar to cancer cells. The patient was an elderly male. Although no other space-occupying lesions were found in whole-body PET-CT scan, the possibility of undetected small cancerous changes could not be ruled out. Therefore, immunohistochemical staining for epithelial cells labeled with CK, CK5/6, CK7, and EMA was performed, and the result was still negative. After excluding inflammatory cells, tissue cells, endothelial cells, and cancer cells, the diagnosis of lymphoma was considered. There are two types of lymphoma that originate from blood vessels: large B-cell lymphoma and NK/T cell lymphoma, but they rarely both originate in the prostate. Immunohistochemical staining showed strong expression of CD20, CD79a, and PAX5 in tumor cells, but CD3, CD56, and TIA1 were not expressed in tumor cells. Moreover, the number of Ki-67-positive cells reached 90%, indicating that the cells in the blood vessels were B-cell lymphoma cells. The patient had no history of lymphoma, and there were no tumor lesions on whole-body PET-CT scans. Furthermore, bone marrow cell tests did not show any tumor cells. Therefore, based on HE staining, microscopy findings, immunohistochemical staining, and overall examination of the patient, we finally consider this case with a rare primary intravascular large B-cell lymphoma originating from the prostate.

If the tumor appears in the skin, central nervous system, bone marrow, liver, or spleen, pathologists can easily diagnose it based on its microscopic morphology. However, when the patient’s main clinical manifestation is prostate hyperplasia, pathologists rarely consider intravascular large B-cell lymphoma. Primary intravascular large B-cell lymphoma of the prostate is extremely rare, and there is currently no genetic, pathogenic, or epidemiological study on primary intravascular large B-cell lymphoma of the prostate. There are also no specific diagnostic indicators for primary intravascular large B-cell lymphoma of the prostate. We provided pathological evidence for more studies on primary intravascular large B-cell lymphoma of the prostate. Pathologists should consider intravascular large B-cell lymphoma when a male patient presents with symptoms of prostate hyperplasia, and large blood vessel cells are present in the background of the enlarged prostate, with round or oval in shape, sparse chromatin, deeply stained nuclei, and diffuse arrangement. As a rare malignant tumor, intravascular large B-cell lymphoma originating from the prostate has always been an important topic in the field of lymphoma research in terms of its pathological characteristics, clinical manifestations, and treatment plans. Although the global incidence rate of IVLBCL is low, it is highly invasive, and it can easily spread to all organs, making early diagnosis and appropriate treatment particularly critical. The existing studies mainly focused on IVLBCL affecting other parts of the body, and there is a lack of case studies with the primary involvement of the prostate, which makes diagnosis and treatment challenging. There are many primary malignant tumors of the prostate, and correct diagnosis and classification play an important role in prognostic evaluation and tumor treatment. Therefore, accurate pathological diagnosis and classification of patients with primary malignant tumors of the prostate pose a huge challenge to pathologists.

## Conclusion

We presented a case report detailing the clinical experience of a patient with primary prostate IVLBCL. Combined with the literature review, we explored the unique pathological phenomena of this disease in the prostate. Intravascular large B-cell lymphoma usually originates from lymph nodes or extranodal lymphoid tissues, and tumors originating from the prostate have been rarely reported globally. This case report provides a detailed explanation of the morphological manifestations, immunophenotypes, and differential diagnosis of IVLBCL in the prostate, providing a reference for diagnosis and treatment. Although chemotherapy is partly effective, further studies are needed to explore the treatment options and prognostic factors for such cases. This case report and literature review can deepen the medical community’s understanding of this rare disease, suggesting that physicians should consider intravascular large B-cell lymphoma when facing atypical prostate diseases. Previous studies on IVLBCL originating from the prostate provide an invaluable reference for clinical diagnosis and highlight challenges in treatment selection and prognosis evaluation. Future studies should focus on the molecular mechanisms of such cases to discover new diagnostic biomarkers and treatment strategies to improve patients’ prognosis and reduce uncertainty in diagnosis and treatment.

## Data Availability

The immunohistochemistry generated during the current study are not publicly available due to ethical privacy but are available from the corresponding author on reasonable request.

## Abbreviations

IVLBCL: Intravascular large B-cell lymphoma
NHL: Non-Hodgkin’s lymphomas
PSA: Prostate-specific antigen
PET-CT: Positron emission tomography-computed tomography
BPH: Benign prostatic hyperplasia‌
ERG: ETS-related gene
TIA1: T-cell intracellular antigen-1
PAX5: Paired box gene-5
